# The Prevalence of Shoulder Disorders among Professional Bullfighters: A Cross-Sectional Ultrasonography Study

**DOI:** 10.3390/tomography8040145

**Published:** 2022-07-04

**Authors:** Álvaro Navas-Mosqueda, Juan Antonio Valera-Calero, Umut Varol, Sebastian Klich, Marcos José Navarro-Santana, César Fernández-de-las-Peñas, Marta Ríos-León, Pedro Belón-Pérez, Eduardo Cimadevilla-Fernández-Pola, Juan Pablo Hervás-Pérez, José Luis Arias-Buría

**Affiliations:** 1 Department of Physiotherapy, Faculty of Health, Camilo José Cela University, Villanueva de la Cañada, 28692 Madrid, Spain; alvaro.navas@ucjc.edu (Á.N.-M.); ecimadevilla@ucjc.edu (E.C.-F.-P.); jphervas@ucjc.edu (J.P.H.-P.); 2 VALTRADOFI Research Group, Department of Physiotherapy, Faculty of Health, Camilo José Cela University, Villanueva de la Cañada, 28692 Madrid, Spain; 1umutvarol7@gmail.com; 3 Faculty of Sports Science, Wrocław University of Health and Sport Science, 51-612 Wrocław, Poland; sebastian.klich@gmail.com; 4 Faculty of Health, Universidad Católica de Ávila (UCAV), 05005 Ávila, Spain; mjose.navarro@ucavila.es; 5 Department of Physical Therapy, Occupational Therapy, Rehabilitation and Physical Medicine, Universidad Rey Juan Carlos, 28922 Alcorcón, Spain; cesar.fernandez@urjc.es (C.F.-d.-l.-P.); joseluis.arias@urjc.es (J.L.A.-B.); 6 Cátedra Institucional en Docencia, Clínica e Investigación en Fisioterapia: Terapia Manual, Punción Seca y Ejercicio Terapéutico, Universidad Rey Juan Carlos, 28922 Alcorcón, Spain; 7 Hospital Nacional de Parapléjicos, Servicio de Salud de Castilla-La Mancha (SESCAM), 45004 Toledo, Spain; mriosl@sescam.jccm.es; 8 Department of Physical Therapy, Real Madrid C.F., 28055 Madrid, Spain; pebelon@gmail.com

**Keywords:** bullfighting, ultrasound imaging, overuse injuries, shoulder, rotator cuff

## Abstract

We aimed to investigate clinical and ultrasound signs of shoulder overuse injuries in professional bullfighters; side-to-side differences (dominant vs. non-dominant); and to determine potential differences according to bullfighters’ categories. An observational cross-sectional study was conducted. Thirty professional and active bullfighters were assessed. A bilateral ultrasound assessment of the subacromial bursa, long biceps head tendon (LHBT), and rotator cuff was performed to determine the presence of bursitis, subluxation, partial or total tendon rupture, tenosynovitis, or calcification. Supraspinatus tendon thickness was measured. Finally, a battery of clinical orthopedic tests (Yergason, Jobe, infraspinatus, Gerber, and bursa tests) were also performed. Most identified ultrasound findings were located in the dominant side, being the presence of bursitis (n = 9; 30%), LHBT tenosynovitis (n = 8; 26.7%), and subscapularis tendon calcification (n = 5; 16.7%) the most prevalent. No side-to-side or between-categories differences were found for supraspinatus tendon thickness (all, *p* > 0.05). The most frequent positive signs were the infraspinatus test (40.0%), Gerber lift-off test (33.3%), and bursitis, Jobe, and Yergason tests (all, 26.7%). Ultrasound signs were commonly found at LHBT, subacromial bursa, and rotator cuff in professional bullfighters without difference between categories and sides. No side-to-side or between-categories differences were found. Positive clinical test signs suggestive of bursitis, LHBT, and rotator cuff tendinopathy were frequently observed.

## 1. Introduction

Bullfighting or tauromachy is a traditional Spanish exhibition actively practiced by 5357 bullfighters divided into seven categories in 2019 according to the Spanish General Registry of Professional Bullfighters [1]. Although the first evidence of the practice is reported in Pamplona in 1385, historical repositories estimate the popularization of bullfighting performance began in 1492 [2,3]. Today, the socio-economic role of bullfighting in Spain is substantial. Recent Spanish statistics registered up to 17,698 events in 2019 [1]. This implies a growing economic impact, since in 2013 tauromachy has generated EUR 1604 million (corresponding to 0.16% of the national product) [4] and the latest data from 2019 showed an increase up to EUR 4100 million and 54,000 job-related positions [5].

Bullfighting is a complex, irregular, and unpredictable physical activity associated with injuries including traumatism, falls, and bull-horn wounds [6,7]. Although there are several reports about the incidence of injuries related to external factors [6,7,8,9,10], current evidence about injuries related to intrinsic factors (e.g., performance biomechanics) is still limited. Previous studies have reported that the most affected joints in non-traumatic injuries are the shoulder, the elbow, and the wrist [6,7,11], being highly associated with the “supreme luck” continuous repetition (the last sword thrust).

Although bullfighting is considered ethically controversial, and a large part of the population dismisses this sport (including Spanish people) because bulls are tortured and killed, there is a need of clinical attention for this population (since according to The Universal Declaration of Human Rights, everyone is entitled to adequate health attention without distinction of any kind) and this article should be understood from a health point of view and not as a bullfighting support. Therefore, although biomechanics and pathophysiology of the shoulder area had been widely assessed for the general population and specific sports [12,13,14], specific bullfighting research assessing non-traumatic injuries is needed.

One of the main limitations stated in previous studies was the lack of accessibility of bullfighters to be assessed due to complicated schedules that require unpredictable and continuous travel. Ultrasound Imaging (US) is a fast, easy, safe, low-cost, and portable imaging method for assessing soft tissue morphology and quality [15], which facilitates quick evaluation in these “unfavorable” situations and provides reliable and valid data in the upper extremity [16]. Therefore, since there are no studies that have previously investigated the shoulder joint of bullfighters, the aims of the current study were: 1, to investigate clinical and ultrasound signs of shoulder overuse injuries in professional bullfighters; 2, to investigate side-to-side differences (dominant vs. non-dominant); and 3, to determine the differences according to bullfighters’ categories.

## 2. Materials and Methods

### 2.1. Study Design

An observational cross-sectional study was conducted. This study followed the Strengthening the Reporting of Observational Studies in Epidemiology (STROBE) guidelines and checklist [17], and was conducted according to the Declaration of Helsinki and supervised by the Institutional Ethics Committee of Universidad Rey Juan Carlos.

### 2.2. Participants

Bullfighters listed in the 4 Bullfighting Schools located in Madrid (Spain) and being active during 2020–2021 were recruited via local announcements between October 2020 and January 2021. Eligibility criteria included being registered and active in Section I or II in the Spanish General Registry of Professional Bullfighters, having a minimum experience of 5 bullfights, and aged from 18 to 45 years old. Exclusion criteria were previous shoulder surgery in the last year, shoulder infiltration in the last year, fractures of any bone related to the shoulder, medication intake affecting the muscle tone, and any other underlying medical condition affecting the study outcomes.

Participants were classified into one of the three following groups according to their bullfighting category: “matador” (the main performer of the entourage and who finally kills the bull) and “picador” (a bullfighter using a special lance designed to prevent deadly injuries, for facilitating the matador information about which side is favored by testing the bull’s strength). This last category is divided into two, riding a horse or without a horse. All subjects provided written informed consent prior to their inclusion.

### 2.3. Assessments

#### 2.3.1. Shoulder Ultrasound Imaging

**Figure 1:**
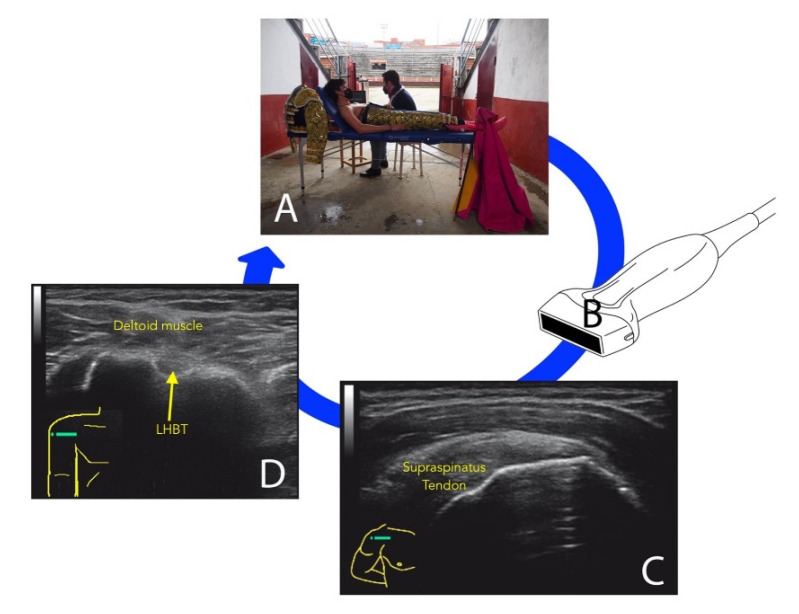
Ultrasound assessment of bullfighters (**A**) with a linear transducer (**B**). Image acquisition of supraspinatus tendon (**C**) and long biceps head tendon (LBHT) (**D**).

Images were acquired with a Vinno G60 (Vinno technology Co., Ltd.; Suzhou, China) US equipment with a 7.3–14 MHz linear probe. Ultrasound imaging is considered a reliable and valid tool for assessing the shoulder since its consistency with magnetic resonance imaging is 71.6%, 95.5%, 83.6%, and 80.6% for supraspinatus, infraspinatus, subscapularis tendons, and long head of the biceps tendon (LHBT), respectively [18,19]. The imaging acquisition procedure was conducted by a single examiner with 5 years of experience following the international standardized guidelines of the European Society of Musculoskeletal Radiology [20]. All images were acquired bilaterally as follows:

The LHBT was examined by positioning participants with their arm slightly externally rotated closed to the chest and the elbow flexed in the longitudinal and transverse plane from the intra-capsular origin to the muscular transition for assessing tendon partial or total rupture, subluxation, and tenosynovitis, as shown in Figure 1.

The rotator cuff was examined for assessing the tendon thickness, partial or total ruptures, bursitis, and calcifications. Rotator cuff tendon tears were indicated by the existence of visible gaps or total absence of tendon tissue in the subacromial space, while calcifications were identified as hyperechoic shapes with posterior acoustic shadows located in the tendon matrix [21]. After examining the LHBT, participants were asked to place the shoulder into a further external rotation for examining the subscapularis tendon between the scapular coracoid process and the humeral lesser tubercle [22]. For examining the supraspinatus tendon, participants were placed in a modified “crass position” with the hand placed on the low back with the elbow flexed, placing the probe in a longitudinal plane for assessing the supraspinatus tendon between the scapular acromial process and the humeral greater tubercle [23]. Subdeltoid bursa was assessed as well in this position; a bursitis diagnosis was considered if thickness was +2 mm [24]. Finally, the imaging of the infraspinatus tendon was made in the same position with the shoulder slightly internally rotated and gliding the transducer to the longitudinal and the transversal planes on the dorsal aspect of the shoulder.

For identifying LHBT subluxation, a dynamic assessment with internal and external rotation was performed; tenosynovitis was identified if the tendon sheath was distended by the presence of hypoechoic or anechoic thickened tissue, with or without local effusion; bursitis was identified as bursal widening, due to an increased amount of synovial fluid with or without synovial hypertrophy; calcifications were identified as hyperechoic structures within the tendon with or without acoustic posterior shadowing; and ruptures were identified as a partial or total break of the tendon fibrillar pattern [25].

#### 2.3.2. Shoulder Clinical Testing

The following battery of orthopedic tests was performed by a different examiner with more than 10 years of experience in assessing the LHBT, the rotator cuff, and bursa.

The Yergason test consists of positioning the participant’s elbow to 90° flexion and forearm pronation, and holding the patient’s wrist, asking the patient to actively supinate against resistance. If the pain is localized into the bicipital groove area, this suggests proximal LHBT tendinopathy. This test has been demonstrated to be the only test of value in diagnosing proximal LHBT pathology with an estimated sensitivity of 0.41 and specificity of 0.84 [26].

The Jobe test is widely used to assess the integrity of the supraspinatus tendon since it showed acceptable (sensitivity: 0.81, specificity: 0.55). Participants were placed in a sitting position with both arms horizontally abducted at 90° and internally rotated at 45°. A collapse event or painful response while applying a downward pressure was considered a positive test [27,28].

The subscapularis tendon was examined with the Gerber lift-off test since it has sensitivity: 1 and specificity: 0.55. The participants started with the dorsum of the hand on the low back in internal rotation and were asked to lift the hand away from the back against the examiner’s resistance. The test was considered positive if the patient could not resist, lift the hand off the back, or compensated by extending the elbow and shoulder [28].

The infraspinatus test (sensitivity: 0.9, specificity: 0.74) consists of evaluating a pain or weakness response while the participant is performing a resisted external rotation from a sited position with the shoulder in a neutral position and elbows flexed 90° [28].

Finally, the bursitis sign is a test (sensitivity: 0.09, specificity: 1) consisting of a manual palpation of the anterolateral subacromial area for determining localized painful tenderness to palpation in the subacromial space [28].

### 2.4. Statistical Analyses

Statistical analyses were performed using the SPSS V.25 software for Mac OS (IBM Corporation, Armonk, NY, USA). All tests were two-tailed with *p*-values < 0.05 considered significant. Normal distribution was verified by using a Shapiro–Wilk test, homogeneity of variance by using a Levene test, and sphericity by using the Mauchly test. A descriptive analysis of the participants is presented as mean and standard deviation for quantitative variables and as frequency numbers and percentages for qualitative variables. Student’s t-tests for independent samples were used to determine dominant versus non-dominant side differences and ANOVA tests were calculated to analyze the effect of the bullfighting category on clinical and US variables. For multiple post hoc comparisons, a Bonferroni correction test (category*age) was applied. Categorical variables were compared across the three groups using Chi-Square tests. Finally, a correlation analysis between sides for US and clinical findings was conducted using Spearman’s Rho.

## 3. Results

A total sample of 30 bullfighters responded to the announcement, none of them were excluded and, therefore, the sample analyzed in this study was 30 males. Participants’ socio-demographic characteristics are described in Table 1. In general, significant age and bullfighting experience differences (*p* < 0.001) were found between categories. Neither body mass index (BMI), height, nor weight showed significant differences (all, *p* > 0.05).

Table 2 shows the results of clinical and US findings regarding shoulder injuries focusing on the rotator cuff, bursa, and LHBT. The most frequent positive signs observed were infraspinatus test (n = 12; 40%), Gerber lift-off test (n = 10; 33.3%), and the bursitis, Jobe and Yergason test (all, n = 8; 26.7%). No differences between the categories were observed (*p* > 0.05). Regarding US alterations, the most common was the presence of bursitis (n = 9; 30.0%), LHBT tenosynovitis (n = 8; 26.7%), and subscapularis tendon calcification (n = 5; 16.7%).

Table 3 shows supraspinatus tendon thickness. In general, the dominant side showed thicker morphology for supraspinatus tendon thickness; however, the difference did not reach statistical significance. Similarly, although bullfighters in the matador category showed thicker supraspinatus tendons compared with both “picador” groups, differences were not statistically significant.

## 4. Discussion

Most of the available evidence regarding shoulder injuries focused on external factors to the bullfighter (e.g., goring or trauma) since up to 89% of injuries needs hospitalization and the mortality reach 0.9% [29,30,31]. A 40-year retrospective study assessing the medical records of bull horn injuries resulted in 572 injuries (14.3 cases/year; 54 of these with multiple injuries), most of them located in the lower limbs, perineum, and abdomen [31]. Another 10-year retrospective report detailed the type of injuries (e.g., open wounds, bruises, fractures, and traumatic brain injuries) and showed a similar incidence (10.7 cases/year) and affected areas (62.4% affected the lower limbs and perineum) [30].

However, to our knowledge, this is the first study focusing on shoulder overuse injuries caused by intrinsic factors related to repetitive loading in bullfighters. Although these injuries are not directly associated with mortality, overuse injuries could affect the bullfighter’s performance. Since a failed performance in professional bullfighters increases the risk of bull horn injury, early identification of these signs and symptoms is important to understand the most prevalent overuse injuries and prevent future damage.

Sports-related tendinopathies are characterized by well-defined histopathological lesions associated with the chronicity of symptoms (including pain and decreased load tolerance and function) [31]. Tendinopathies may result from an ineffective healing process of repeated micro-injuries produced because of continuous heavy loading, training errors (e.g., poor technique or inadequate equipment), or soft tissue status [32]. Further, several intrinsic factors including previous injury, insufficient or excessive range of motion, rotator cuff weakness, years of athletic practice, body mass index, sex, age, and level of practice are shown to be intrinsic factors associated with risk of injury, which is potentially modifiable by preventive programs [33].

Although bullfighting performance is characterized by different kinematics, kinetics, and general muscle activity depending on the category, our results showed similar clinical shoulder overuse findings in all categories. Likely to baseball pitching and other overhead sports, e.g., tennis, swimming, or volleyball, the LHBT was affected (mostly tenosynovitis and subluxation on the dominant side) [34]. Bursitis was also moderately found in our sample. The subacromial-subdeltoid bursa normally contains a trace volume of fluid to minimize the friction during supraspinatus movement and its inflammation can often occur with overuse shoulder conditions including acromioclavicular osteoarthritis and supraspinatus tendon tear, calcification, acute trauma, and rheumatoid arthritis [35].

Finally, the rotator cuff comprises the supraspinatus, infraspinatus, teres minor, and subscapularis tendons. Although rotator cuff degeneration and tearing are the most common findings in overhead athletes [36], our results showed a low percentage of rotator cuff total or partial ruptures in professional bullfighters, with no side-to-side differences or between-categories differences in supraspinatus tendon thickness. This low prevalence rate may be related to the fact that bullfighting is not associated with overhead shoulder movements as other sports such as handball or swimming.

Although this is the first study investigating the shoulder area in professional bullfighters, some limitations should be recognized. First, the sample size was limited. Therefore, our results should not be considered as potential normative data, and future studies are needed to determine a more accurate intrinsic injury prevalence. In addition, we did not include a control group not practicing bullfighting. For this reason, it is not possible to determine a cause-and-effect contribution of bullfighting to the prevalence of shoulder injuries observed in the current study.

## 5. Conclusions

This study found that LHBT tenosynovitis and subluxation, bursitis, and rotator cuff calcifications (supraspinatus: 3.3%, infraspinatus: 10%, and subscapularis: 16.7%) were the most prevalent ultrasound findings in professional bullfighters. No side-to-side or between-category differences were observed for supraspinatus tendon thickness. Positive clinical tests for bursa (26.7%), LHBT (26.7%), supraspinatus (26.7%), infraspinatus (40%), and subscapularis (33%) tendons were also present.

## Figures and Tables

**Table 1:** Socio-demographic Characteristics of the Sample.

Variables	Matador (n = 10)	Picador with Horse (n = 10)	Picador without Horse (n = 10)
Dominant side (Right/Left, n)	10/0	10/0	10/0
Experience (years) *	16.1 ± 7.2	5.3 ± 1.4	2.8 ± 1.0
Age (years) *	31.3 ± 7.6	22.7 ± 3.2	19.9 ± 1.6
Height (meters)	1.78 ± 0.08	1.80 ± 0.04	1.75 ± 0.06
Weight (kg)	69.7 ± 8.1	69.3 ± 5.6	66.1 ± 5.6
BMI (kg/m^2^)	21.8 ± 1.5	21.3 ± 1.2	21.4 ± 0.7

Values are expressed as mean ± SD. * Significant differences between categories (*p* < 0.001).

**Table 2:** Clinical and Ultrasonographic Tests.

Variables	Matador (n = 10)		Picador with Horse (n = 10)		Picador without Horse (n = 10)		Chi-Squared (between-Categories)		Spearman’s Rho (between-Sides)
	Dominant	Non-Dominant	Dominant	Non-Dominant	Dominant	Non-Dominant	Dominant	Non-Dominant	
*Long Head Biceps Tendon*									
Tenosynovitis (Y/N; n, %)	3/7; 30.0/70.0	0/10; 0.0/100.0	3/7; 30.0/70.0	0/10; 0.0/100.0	2/8; 20.0/80.0	0/10; 0.0/100.0	0.843	-	0.392; *p* = 0.002
Subluxation (Y/N; n, %)	1/9; 10.0/90.0	0/10; 0.0/100.0	2/8; 20.0/80.0	0/10; 0.0/100.0	0/10; 0.0/100.0	0/10; 0.0/100.0	0.329	-	0.229; *p* = 0.078
Rupture (Y/N; n/%)	0/10; 0.0/100.0	0/10; 0.0/100.0	0/10; 0.0/100.0	0/10; 0.0/100.0	0/10; 0.0/100.0	0/10; 0.0/100.0	-	-	-
Yergason Test (Y/N; n, %)	3/7; 30.0/70.0	1/9; 10.0/90.0	3/7; 30.0/70.0	0/10; 0.0/100.0	1/9; 10.0/90.0	0/10; 0.0/100.0	0.475	0.355	0.294; *p* = 0.023
*Supraspinatus Tendon*									
Calcification (Y/N; n, %)	1/9; 10.0/90.0	0/10; 0.0/100.0	0/10; 0.0/100.0	0/10; 0.0/100.0	0/10; 0.0/100.0	0/10; 0.0/100.0	0.355	-	0.130; *p* = 0.321
Rupture (Y/N; n/%)	0/10; 0.0/100.0	0/10; 0.0/100.0	1/9; 10.0/90.0	0/10; 0.0/100.0	0/10; 0.0/100.0	0/10; 0.0/100.0	0.355	-	0.129; *p* = 0.330
Jobe Test (Y/N; n, %)	2/8; 20.0/80.0	2/8; 20.0/80.0	2/8; 20.0/80.0	1/9; 10.0/90.0	1/9; 10.0/90.0	0/10; 0.0/100.0	0.787	0.329	0.098; *p* = 0.456
*Infraspinatus Tendon*									
Calcification (Y/N; n, %)	0/10; 0.0/100.0	2/8; 20.0/80.0	1/9; 10.0/90.0	0/10; 0.0/100.0	0/10; 0.0/100.0	0/10; 0.0/100.0	0.355	0.117	−0.076; *p* = 0.561
Rupture (Y/N; n/%)	0/10; 0.0/100.0	0/10; 0.0/100.0	0/10; 0.0/100.0	0/10; 0.0/100.0	0/10; 0.0/100.0	0/10; 0.0/100.0	-	-	-
Infraspinatus Test (Y/N; n, %)	3/7; 30.0/70.0	2/8; 20.0/80.0	5/5; 50.0/50.0 *	0/10; 0.0/100.0 *	1/9; 10.0/90.0	1/9; 10.0/90.0	0.149	0.329	0.250; *p* = 0.054
*Subscapularis Tendon*									
Calcification (Y/N; n, %)	3/7; 30.0/70.0	0/10; 0.0/100.0	1/9; 10.0/90.0	0/10; 0.0/100.0	1/9; 10.0/90.0	0/10; 0.0/100.0	0.383	-	0.302; *p* = 0.019
Rupture (Y/N; n/%)	0/10; 0.0/100.0	0/10; 0.0/100.0	0/10; 0.0/100.0	0/10; 0.0/100.0	0/10; 0.0/100.0	0/10; 0.0/100.0	-	-	-
Gerber Test (Y/N; n, %)	2/8; 20.0/80.0	1/9; 10.0/90.0	4/6; 40.0/60.0 *	0/10; 0.0/100.0 *	2/8; 20.0/80.0	1/9; 10.0/90.0	0.506	0.585	0.268; *p* = 0.038
*Bursa*									
Bursitis (Y/N; n, %)	3/7; 30.0/70.0	1/9; 10.0/90.0	2/8; 20.0/80.0	2/8; 20.0/80.0	0/10; 0.0/100.0	1/9; 10.0/90.0	0.186	0.749	0.047; *p* = 0.723
Bursa Test (Y/N; n, %)	3/7; 30.0/70.0	1/9; 10.0/90.0	3/7; 30.0/70.0	1/9; 10.0/90.0	0/10; 0.0/100.0	0/10; 0.0/100.0	0.153	0.585	0.196; *p* = 0.133

* Significant differences between dominant and non-dominant sides (*p* < 0.05).

**Table 3:** Supraspinatus Tendon Ultrasonographic Characteristics.

Variables	Mean	Dominant Side	Non-Dominant Side	Between-Sides Difference
*Supraspinatus Tendon Thickness*				
Matador	0.58 ± 0.06	0.60 ± 0.07	0.55 ± 0.07	0.05 (−0.02;0.12) *p* = 0.16
Picador with horse	0.55 ± 0.07	0.56 ± 0.08	0.53 ± 0.06	0.03 (−0.03;0.10) *p* = 0.31
Picador without horse	0.53 ± 0.06	0.54 ± 0.06	0.53 ± 0.06	0.00 (−0.05;0.06) *p* = 0.96
Weight (kg)	69.7 ± 8.1	69.3 ± 5.6	66.1 ± 5.6	
*Between-Categories Differences*				
ANOVA (Category*Age)	F = 2.59; *p* = 0.12	F = 2.64; *p* = 0.12	F = 1.54; *p* = 0.28	
Matador-Picador with horse	0.03 (−0.04;0.10) *p* = 0.60	0.03 (−0.05;0.12) *p* = 0.69	0.02 (−0.05;0.10) *p* = 0.88	
Matador-Picador without horse	0.04 (−0.03;0.11) *p* = 0.29	0.06 (−0.02;0.15) *p* = 0.17	0.01 (−0.05;0.09) *p* = 1.00	
Picador with-without horse	0.01 (−0.06;0.08) *p* = 1.00	0.02 (−0.06;0.11) *p* = 1.00	0.00 (−0.08;0.07) *p* = 1.00	

Descriptive Values are expressed as Mean ± Standard Deviation. Difference values are expressed as Mean (95% CI).

## Data Availability

Not applicable.
